# Molecular Resonance Identification in Complex Absorbing Potentials via Integrated Quantum Computing and High-Throughput Computing

**DOI:** 10.1021/acs.jctc.5c01939

**Published:** 2026-03-09

**Authors:** Jingcheng Dai, Atharva Vidwans, Eric H. Wan, Alexander X. Miller, Micheline B. Soley

**Affiliations:** † Department of Chemistry, 5228 University of Wisconsin-Madison, 1101 University Avenue, Madison, Wisconsin 53706, United States; ‡ Department of Physics, University of Wisconsin-Madison, 1150 University Avenue, Madison, Wisconsin 53706, United States; § Department of Mathematics, University of Wisconsin-Madison, 480 Lincoln Drive, Madison, Wisconsin 53706, United States; ∥ Department of Computer Sciences, University of Wisconsin-Madison, 1210 West Dayton Street, Madison, Wisconsin 53706, United States; ⊥ Data Science Institute, University of Wisconsin-Madison, 447 Lorch Street, Madison, Wisconsin 53706, United States; 6 Woodbridge High School, 2 Meadowbrook, Irvine, California 92604, United States

## Abstract

Recent advancements in quantum algorithms have reached a state where we can consider how to capitalize on quantum and classical computational resources to accelerate molecular resonance state identification. Here, we identify molecular resonances with a method that combines quantum computing with classical high-throughput computing (HTC). This algorithm, which we term qDRIVE (the quantum deflation resonance identification variational eigensolver), exploits the complex absorbing potential formalism to distill the problem of molecular resonance identification into a network of hybrid quantum-classical variational quantum eigensolver tasks and harnesses HTC resources to execute these interconnected but independent tasks both asynchronously and in parallel, a strategy that minimizes wall time to completion. We show qDRIVE successfully identifies resonance energies and wave functions in simulated quantum processors with current and planned specifications, which bodes well for qDRIVE’s ultimate application in disciplines ranging from photocatalysis to quantum control and places a spotlight on the potential offered by integrated heterogeneous quantum computing/HTC approaches in computational chemistry.

## Graphical abstract

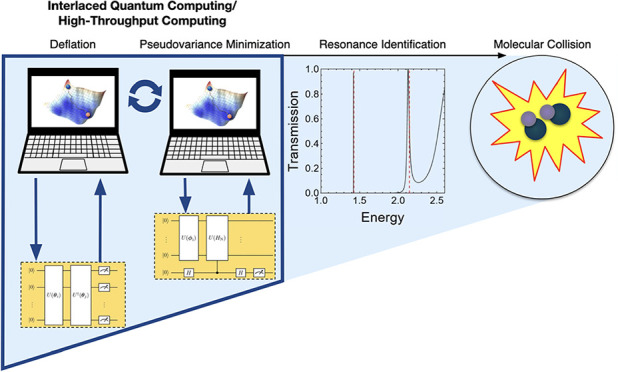

## Introduction

To date, much of the focus on quantum algorithms in chemistry has been placed on identification of the ground state and excited states of Hermitian Hamiltonians.^1–17^ However, an emerging interest lies in open quantum systems, both to simulate realistic chemical systems in contact with surrounding environments^18^ and to characterize physical systems—including molecular qubits—to use as the basis of novel quantum computing architectures.^19^ The newfound interest in open quantum systems has led to the development of quantum algorithms to identify eigenstates of associated non-Hermitian Hamiltonians,^20–29^ including left- and right-eigenvectors associated with parity-time reversal (PT) symmetry breaking at exceptional points^28,29^ and resonances (purely outgoing eigenstates) associated with decay processes.^21,24,27,30^

Quantum algorithms for resonance identification are of particular relevance in today’s computational chemistry since resonances are ubiquitous for the description of molecular breakup processes in chemistry ranging from ultracold collision complex decay^31^ to plasmonic photocatalysis.^32^ Recent predictions also suggest molecular resonances could play a key role in the ongoing second quantum revolution with impacts to both quantum information processing^33^ and quantum control.^34^ Many classical methods to identify resonances exist, such as approaches based on complex absorbing potentials (CAPs),^35–39^ complex scaling,^40,41^ Feshbach projection,^42–44^ the stabilization method,^45–47^ analytic continuation in the coupling constant,^48,49^ and R-matrix theory.^50^ However, contemporary methods for molecular resonance identification often struggle with exacting parameter dependence and/or sizable basis sets.^32^ This begets the question: how can we harness available computational resources—both quantum and classical—to facilitate and accelerate molecular resonance identification?

Current quantum algorithms that address this question tend to fall into two categories.[Fn fn1] The first category comprises algorithms based on quantum phase estimation. This category can be considered to include algorithms such as measurement-based quantum phase estimation,^20^ iterative phase estimation,^21^ generalizations of quantum phase estimation,^22,23,25^ the iterative Harrow-Hassidim-Lloyd approach,^52^ and the direct measurement method.^27,53,54^ Since quantum phase estimation tends to call for a high circuit depth (gate count), algorithms in this category are often best suited for fault-tolerant quantum computers still under development. The second category comprises algorithms that adapt the variational quantum eigensolver (VQE)^3,55–58^ to non-Hermitian systems. This category can be considered to include algorithms such as the Variational Quantum Universal Eigensolver^26^ and Xie-Xue-Zhang method.^28–30^ Since these algorithms rely on a VQE-like approach, algorithms in this category typically require lower circuit depths than algorithms based on quantum phase estimation, and thus are more naturally suited to existing noisy intermediate scale quantum (NISQ) computers. The adaptation of VQE to non-Hermitian systems entails an added computational cost related to preparation of the Ansatz (trial or guess state); for example, the Variational Quantum Universal Eigensolver requires preliminary precise training^26^ and the Xie-Xue-Zhang method requires both parametrization of and scans over the eigenenergy.^28^

In this study, we facilitate molecular resonance identification by combining the complex absorbing potential (CAP) formalism with an interlaced quantum computing/parallel asynchronous high-throughput computing (HTC) approach that we term qDRIVE (the quantum deflation resonance identification variational eigensolver).

We recognize that the construction of CAPs enables us to prepare a physically motivated Ansatz without precise training or energy parametrization as follows: In the CAP formalism,^32,35–39,59,60^ resonances are identified by appending to the original potential of interest *V*_0_ a negative imaginary potential at the outer reaches of the simulation window i*V*_CAP_ to impose purely outgoing boundary conditions. The CAP formalism thus entails two Hamiltonians: a Hermitian Hamiltonian *H*_H_ comprised of the system’s physical kinetic and potential energy and an artificial non-Hermitian Hamiltonian that is only made non-Hermitian by the imposition of boundary conditions with a CAP *H*_N_. Since the two Hamiltonians differ only at the extremities of the simulation window, their eigenstates are, in many systems, likely to be similar in the internal region. Therefore, an effective initial guess for an eigenstate of the non-Hermitian Hamiltonian is often the corresponding eigenstate of the Hermitian Hamiltonian. qDRIVE uses this strategy to prepare the initial Ansatz for the non-Hermitian Hamiltonian resonance as the corresponding eigenstate of the Hermitian Hamiltonian. This identification of the required Hermitian Hamiltonian eigenstates is a straightforward task on quantum computers with VQE^3,55–58^ and its excited-state analog, variational quantum deflation (VQD).^10^

Importantly, CAP-based qDRIVE provides the means to sidestep many of the complexities involved in non-Hermitian quantum mechanics. Specifically, in Hermitian quantum mechanics, wave functions are decomposed in terms of the complete set of eigenfunctions of a Hermitian operator, where orthogonality is determined according to the standard scalar product ⟨ψ|ϕ⟩ = ∫d*x*ψ^★^(*x*)­ϕ­(*x*). In non-Hermitian quantum mechanics, decomposition of wave functions instead requires the c-product given by (ψ|ϕ) = ∫d*x*ψ­(*x*)­ϕ­(*x*).^61,62^ Here a significant complication arises–eigenvalues of a non-Hermitian operator can coalesce, as often occurs in parity-time-reversal-symmetric (PT) systems^63–65^ and in complex scaling at the complex rotation angle where continuum and resonance solutions meet.^66^ In this case, eigenvalues become defective and exceptional points appear; and eigenfunctions themselves coalesce such that the spectrum becomes incomplete or defective. Simulation methods must then contend with self-orthogonality, in which the c-product becomes zero, and an accompanying lack of biorthogonal completeness. Such a situation typically calls for special adaptations such as the inclusion of functions beyond true eigenfunctions in order to ensure closure relations hold.^67^

CAP-based resonance identification in qDRIVE allays these concerns at two levels: First, at the algorithmic level, by construction, qDRIVE never calls for calculation of the c-product, only the standard scalar product, for which these problems do not occur. Second, at the NISQ computing level, inherent quantum processor error is expected to break apart degeneracies. It is well-known that an infinitesimal perturbation is sufficient to destroy an exceptional point: for example, round-off error is sufficient to avoid a zero c-product in finite-element approaches.^66,67^ By definition, NISQ computers similarly involve a significant (typically larger) degree of error, both in terms of thermal and gate noise and in terms of limitations on the gate count that lead to finite Ansatz expressibility. Just as round-off error pushes systems away from the exceptional point on classical processors, NISQ systems’ error is similarly expected to drive systems away from exact exceptional points to allow for the restoration of biorthogonal completeness.

Today, there is active discussion on how to employ high-performance computing (HPC) resources to accelerate quantum information processing tasks.^68–71^ Traditionally, such HPC approaches accelerate computation by running blocks of jobs in parallel, often with tight communication between jobs. However, where HPC resources are limited, such an approach can require large wait times until a block of a suitable size becomes available. In contrast, HTC follows a vulture’s approach that scavenges individual available computational cores as they become available to run jobs—still in parallel, but now asynchronously as resources allow.^72–78^ This HTC approach maximizes throughput of computational tasks performed by the supercomputer over time, an important tack where supercomputing resources are scarce. qDRIVE consists of a network of interconnected but independently executable jobs. This network can be readily expressed as a directed acyclic graph, which can be implemented on HTC resources with HTCondor DAGMan (the HTCondor directed acyclic graph manager).^72–78^

Typically, HTCondor DAGMan uses fall into three main categories based on the instructed order of task operations: (1) sequential DAGs, in which task A is completed prior to task B, task B prior to task C, and so on; (2) split-and-recombine DAGs, in which a task begets many tasks that feed into a single shared task; and (3) collection DAGs, in which there is no ordering of tasks to be completed.^79^ qDRIVE uses a DAG that falls outside these categories. This DAG can be described rigorously as an arborescence in which each parent node leads to a child node of the same connectivity and a child node with no children. Such an interlaced DAG hybridizes the cascading nature of a sequential DAG with the spawning ability of a split-and-recombine DAG. In addition, the DAG itself is executed in separate batch runs, such that the DAG also shares the embarrassingly parallel characteristic nature of a collection DAG. This innovative hybrid DAG approach accelerates completion of all of qDRIVE’s interdependent Hermitian and non-Hermitian Hamiltonian stages by closely mirroring qDRIVE’s algorithmic structure and the execution of tasks with the DAGMan metascheduler.

To the knowledge of the authors, the framework introduced in qDRIVE is the only framework to harness HTC resources in quantum computing for chemical applications to date in the literature. The only known use of comparable high-throughput computing systems in quantum computing is the use of HTCondor to simulate quantum computers classically,^80^ which constitutes a distinct goal from the acceleration of hybrid quantum-classical algorithms that may employ real quantum computers as put forward here. The HTC framework introduced in qDRIVE is therefore expected to be of unique benefit to researchers seeking to accelerate completion of hybrid quantum-classical algorithm jobs, as well as to those who have access to publicly available HTC resources such as the Open Science Grid but who may have only limited access to more widely known HPC resources.^81,82^

In proof-of-concept experiments using a variety of quantum simulators, we show qDRIVE identifies the bound and resonance states of a long-established benchmark model of molecular predissociation^53,62^ (i) in the zero-noise limit and (ii) on NISQ systems in conjunction with the practical error mitigation techniques^83,84^ of readout matrix inversion^85–87^ and hybrid linear-exponential zero-noise extrapolation (ZNE).^83,88–91^ For the same system, we also show qDRIVE fares well (iii) in far-term environments, modeled by the anticipated gate errors and qubit longevities of quantum processors on the horizon. These successes show that qDRIVE can be used to identify molecular resonance states capitalizing on a joint quantum computing and HTC heterogeneous computing approach.

## Methods

### qDRIVE Algorithmic Framework

As the basis of a quantum algorithm for molecular resonance identification in the complex absorbing potential (CAP) formalism,^32,35–39,59,60^ we consider the class of Siegert pseudostates defined as the purely outgoing eigenstates of a non-Hermitian Hamiltonian *H*_N_ given by the sum of (i) the Hermitian Hamiltonian *H*_H_ corresponding to the purely real potential *V*_0_ of interest and (ii) a complex absorbing potential (CAP) *V*_CAP_

1 $$H_{N}=H_{H}+iV_{\mathrm{CAP}}$$

where *V*_CAP_ is purely a real potential that is zero in the internal region of physical interest and negative near the simulation boundary in order to impose purely outgoing boundary conditions. Eigenstates of the resulting non-Hermitian Hamiltonian are complex in energy *E* = *E*_r_ – i*E*_i_ with an imaginary part that corresponds to the resonance decay rate *E*_i_ = Γ/2 and lifetime τ = Γ^–1^. Note practical identification of physical Siegert states *H*_N_ψ = *E*ψ further requires omission of spurious resonance states that correspond to artifacts native to the CAP method (namely, nonresonant, diverging, and indifferent states, see ref 92).

To characterize resonances in this CAP formalism, we consider the purely nonnegative metric for a Hamiltonian H_O_ of

2 $$\sigma_{\mathrm{pseudo}}^{2}=\left\langle H_{O}^{†}H_{O}\right\rangle -\left\langle H_{O}^{†}\right\rangle \left\langle H_{O}\right\rangle$$

where the subscript O = {H, N} denotes the Hermiticity or non-Hermiticity of the Hamiltonian, respectively, and where we refer to the metric as the pseudovariance to distinguish the metric from the standard, simplified variance formula for Hermitian Hamiltonians O = H used in standard Variance VQE^93^ and related variance-based quantum algorithms for excited state determination such as the contracted quantum eigensolver (CQE),^15,94^ VQE-X,^95^ CoVaR,^96^ and excited-state variance VQE,^97,98^ namely

3 $$\sigma^{2}=\left\langle H_{O}^{2}\right\rangle -{\left\langle H_{O}\right\rangle }^{2}$$

for O = H, which yields a nonordered field if applied to generic non-Hermitian matrices O = N. This pseudovariance metric assumes the same form as the squared-residual cost function used in variance VQE to identify right eigenvectors of non-Hermitian Hamiltonians^28^ with the key exception that the pseudovariance analytically eliminates dependence on an energy parameter. Note that, since the pseudovariance obviates the need for an energy parameter, use of the pseudovariance circumvents the parameter scan required by prior non-Hermitian Variance VQE approaches.^28–30^ Furthermore, since the CAP-based Hamiltonian *H*_N_ eq 1 is given by the sum of a Hermitian Hamiltonian *H*_H_ and a purely imaginary term i*V*_CAP_, its expectation value ⟨*H*_N_⟩ follows directly from the expectation values of two Hermitian operators, namely, ⟨*H*_N_⟩ = ⟨*H*_H_⟩ + i⟨*V*_CAP_⟩. This decomposition allows the expectation value of the energy of a resonance to be computed using only the scalar product^92^ ⟨ψ|ϕ⟩= ∫d*x*ψ^★^(*x*)­ϕ­(*x*), without recourse to the standard c-product used for generic non-Hermitian systems (ψ|ϕ) = ∫d*x*ψ­(*x*)­ϕ­(*x*).^61,62^ Similar arguments hold for the remaining terms of the pseudovariance ⟨*H*_N_^†^⟩ and ⟨*H*_N_^†^ *H*_N_⟩ such that (i) the pseudovariance may be computed using only the standard scalar product and thereby its associated quantum circuits^3,58^ without recourse to twin pairs of left- and right-eigenvector Ansatz parameters^28,29^ and (ii) the pseudovariance can be shown to be purely nonnegative and to feature global minima of zero only for eigenstates, i.e.

4 $$\sigma_{\mathrm{pseudo}}^{2}\geq 0$$

where σ_pseudo_^2^ = 0 for states ψ that satisfy *H*_N_ψ = *E*ψ.

These characteristics of the pseudovariance, combined with the aforementioned close relationship between *H*_N_ and *H*_H_ in the CAP formalism, enable the identification of *N* eigenstates of *H*_N_ according to the scheme depicted in Figure 1 and outlined below:

1. Consider a parametrized Ansatz wave function ψ­(**θ**) subject to the CAP-based Hamiltonian *H*_N_ = *H*_H_ + i*V*_CAP_ and initialize *i* = 1 and a set of random parameters {**θ**_*i*_}.
2. For the Hermitian Hamiltonian *H*_H_, determine the Ansatz parameters **θ**_*i*_ that correspond to the *i*th eigenstate via optimization of the objective function
5 $$\left\langle \psi \left(\theta_{i}\right)\left|H_{H}\right|\psi \left(\theta_{i}\right)\right\rangle +c\sum_{j<i}{\left|\left\langle \psi \left(\theta_{i}\right)|\psi \left(\theta_{j}\right)\right\rangle \right|}^{2}$$
  for penalty parameter *c*.
3. Store the optimized Ansatz parameters **θ**_*i*_, initialize **ϕ**_*i*_ ≔ θ_*i*_, and increment *i* → *i* + 1. In parallel:
  (a) Return to step 2, AND
  (b) For the non-Hermitian Hamiltonian *H*_N_, determine the Ansatz parameters **ϕ**_*i*_ that correspond to the *i*th eigenstate via optimization of the pseudovariance objective function
6 $$\left\langle \psi \left(\phi_{i}\right)\left|H_{N}^{†}H_{N}\right|\psi \left(\phi_{i}\right)\right\rangle -{\left|\left\langle \psi \left(\phi_{i}\right)\left|H_{N}\right|\psi \left(\phi_{i}\right)\right\rangle \right|}^{2}$$
4. Proceed until *i* = *N* + 1.

**Figure 1:**
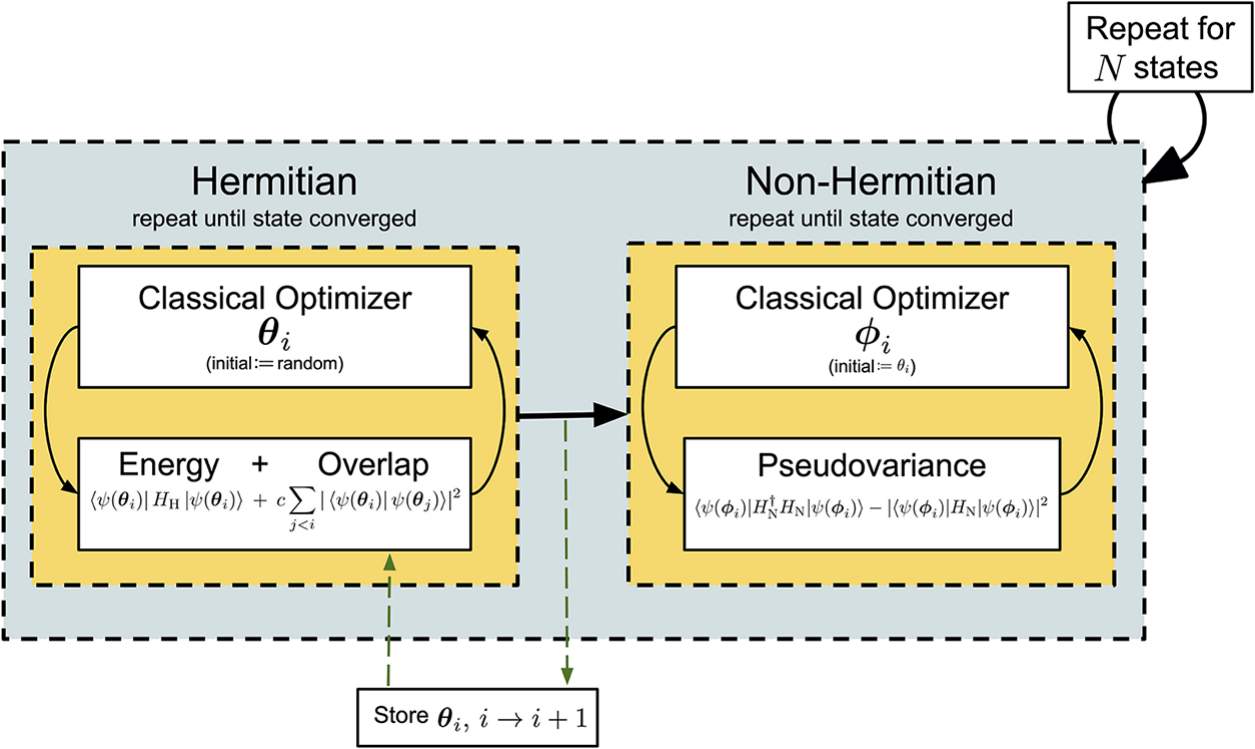
Schematic of the qDRIVE algorithm.

### Hybrid Quantum-Classical Implementation

To implement the aforementioned qDRIVE algorithm as a hybrid quantum-classical approach, we begin by generating the Ansatz wave function as a quantum circuit according to conventional VQE techniques,^55–58^ in which the Ansatz wave function consists of a series of unitary gates parametrized by *m* angles **θ**_*i*_ for eigenstate *i*, |ψ­(**θ**_*i*_)⟩ = *U*(**θ**_*i*_)|0̅⟩ = *U*(θ_*i*_*m*__)*U*(θ_*i*_*m*–1__) ...*U*(θ_*i*_1__)|0̅⟩, where the form of the Ansatz is chosen to sufficiently balance expressibility and circuit depth. To meet these requirements, here we employ the three-layer efficient SU(2) Ansatz depicted in Figure 2(a) with parameters initialized as pseudorandom angles in the domain θ_*i*_*o*__ ∈ [−π,π] for *o* ∈ [1, *m*].

**Figure 2:**
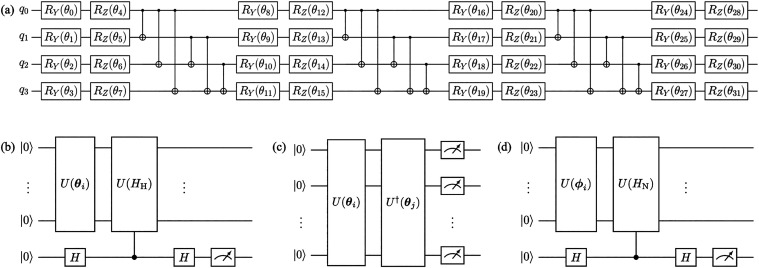
Quantum circuits for (a) the three-layer efficient SU(2) Ansatz, (b) the Hadamard test for estimation of the expectation value of Pauli word components of the Hermitian Hamiltonian Re­(⟨*U*(*H*_H_)⟩), (c) the overlap between two states ⟨ψ­(**θ**_*j*_)|ψ­(**θ**_*i*_)⟩ = ⟨0̅|*U* ^†^(**θ**_*j*_)*U*(**θ**_*i*_)|0̅⟩, and (d) the Hadamard test for estimation of the expectation value Pauli word components of the non-Hermitian Hamiltonian Re­(⟨*U*(*H*_N_)⟩).

Eigenstate identification for the Hermitian Hamiltonian *H*_H_ is then performed via variational quantum deflation (VQD),^10^ in which the ground state and successively identified eigenstates are determined via VQE of an iteratively deflated Hamiltonian. Specifically, the Hermitian Hamiltonian *H*_H_ is represented as a weighted sum of a set of unitary terms amenable to encoding on a *q*-qubit quantum processor, here the *q*-qubit Pauli decomposition

7 $$M=\sum_{k_{1}=1}^{4}\sum_{k_{2}=1}^{4}\cdot \cdot \cdot \sum_{k_{q}=1}^{4}C_{k_{1},k_{2},...,k_{q}}P_{k_{1},k_{2},...,k_{q}}$$

8 $$C_{k_{1},k_{2},...,k_{q}}=\mathrm{Tr}\left(MP_{k_{1},k_{2},...,k_{q}}\right)$$

9 $$P_{k_{1},k_{2},...,k_{q}}=s_{k_{1}}⊗s_{k_{2}}⊗\cdot \cdot \cdot ⊗s_{k_{q}}$$

where *P*_*k*_1_,*k*_2_, ...,*k*_*q*__ denotes a Pauli word comprised of Pauli gates *s*_*k*_*l*__ ∈ {*I*, *X*, *Y*, *Z*} for *j* = 1, 2, ..., *q*

10 $$I=\left[\begin{array}{ll} 1 & 0\\ 0 & 1 \end{array}\right],X=\left[\begin{array}{ll} 0 & 1\\ 1 & 0 \end{array}\right],Y=\left[\begin{array}{ll} 0 & -i\\ i & 0 \end{array}\right],Z=\left[\begin{array}{ll} 1 & 0\\ 0 & -1 \end{array}\right]$$

The objective function eq 5 is then estimated on the quantum processor using the decomposition at values of **θ**_*i*_ selected by a classical optimizer to converge toward the optimal values associated with the Hermitian Hamiltonian eigenstates: The classically expensive tasks of expectation value ⟨ψ­(**θ**_*i*_)|*H*_H_|ψ­(**θ**_*i*_)⟩ and overlap |⟨ψ­(**θ**_*i*_)|ψ­(**θ**_*j*_)⟩|^2^ estimation are performed on the quantum processor via the Hadamard test shown in Figure 2(b) or a direct product of qubit measurements in the basis of each Pauli word for the former and the SWAP test,^99,100^ the destructive SWAP test,^101,102^ or the low-depth overlap method^10,103^ (shown in Figure 2(c)) for the latter; and the classically efficient task of optimization is performed on the classical processor, ensuring the choice of classical optimizer suits the degree of noise associated with the quantum measurements and the choice of a penalty parameter *c* weighs sufficient deflation of the Hamiltonian with ease of the optimization.^13^ According to these considerations, here we employ a penalty parameter of *c* = 100 with 2^9^ maximum optimization iterations for all classical optimizations, using COBYLA^104^ (Constrained Optimization BY Linear Approximation, initial variable change *P*_beg_ = 1) in the absence of noise and the NFT method^105^ (the Nakanishi-Fujii-Todo method, maximum function evaluations *f*_max_ = 2^11^ and reset interval *R* = 32) in the presence of noise.

To converge the parameters associated with the Hermitian Hamiltonian eigenstates **θ**_*i*_ to the parameters associated with the non-Hermitian Hamiltonian eigenstates **ϕ**_*i*_, we employ a hybrid quantum-classical approach to pseudovariance optimization. We decompose the operators required for evaluation of the pseudovariance objective function eq 6 *H*_N_ and *H*_N_^†^ *H*_N_ according to the Pauli decomposition eq 7, employ the quantum processor to estimate their expectation values (see example in Figure 2(d)), and identify the optimal parameters **ϕ**_*i*_ using a classical optimizer suitable in both noiseless and noisy conditions, here Py-BOBYQA^106,107^ (Bound Optimization BY Quadratic Approximation in Python, maximum function evaluations *f*_max_ = 2^10^, requested maximum pseudovariance tolerance *f*_tol_ = 0.05, maximum full retrials to reach tolerance *u* = 3, and initial trust region radius *R*_beg_ = 1).

To reduce variance between instances of qDRIVE and thereby increase the robustness of the algorithm, the aforementioned procedure is executed as a batch of B runs (here B=8 ). Duplicate states identified within a single run are omitted where the overlap between states is above a set tolerance, and the *i*th eigenstate of *H*_N_ with the lowest value of the pseudovariance across batches is considered the optimal representative of the state. Nonresonant, diverging, and indifferent states^92^ are then omitted through analysis of the eigenspectrum to produce the terminal list of resonances.

Given the asynchronicity of and minimal communication between the aforementioned Hermitian and non-Hermitian Hamiltonian eigenstate identification tasks, high-throughput computing (HTC)^72–78^ is used to minimize wall time to completion of the qDRIVE algorithm, with underlying classical computing tasks executed in parallel according to the directed acyclic graph illustrated in Figure 3. For each run, one classical processor identifies the ground state of the Hermitian Hamiltonian ψ­(**θ**_1_), which initiates identification of the first eigenstate of the non-Hermitian Hamiltonian ψ­(**ϕ**_1_) on one processor and identification of the first excited state of the Hermitian Hamiltonian ψ­(**θ**_2_) on another. Convergence of the first excited state of the Hermitian Hamiltonian ψ­(**θ**_2_) then spurs identification of the second excited state of the non-Hermitian Hamiltonian ψ­(**ϕ**_2_) and identification of the second excited state of the Hermitian Hamiltonian ψ­(**θ**_3_) and so on, incrementing *i* ≔ *i* + 1 and proceeding until all *N* eigenstates of *H*_N_ have been identified. The set of *N* eigenstates of *H*_N_ for each run {ψ­(**ϕ**_*i*_)} are then communicated to a single processor per run to pool information and finally on a single processor per batch to perform postprocessing and visualization. The opportunity for parallelism offered by the directed acyclic graph may be paired with further parallelisms presented by the structure of the system under study. For example, where both the potential of interest *V*_0_ and CAP *V*_CAP_ are of shared even parity, the eigenstates they support must also be of well-defined parity such that even and odd states may be constructed independently; where applicable, this parallelism (i) reduces the number of basis states that must be represented on the quantum processor for a given accuracy by half, which amounts to a reduction of the qubit count by one, and (ii) enables classical tasks to be divided into two identical copies of the same directed acyclic graph (one for each basis, odd and even), which reduces the wall time by half. These wall time improvements are obtained at the trade-off of a larger number of quantum circuit evaluations and classical processors, respectively. Additionally, this divided consideration of even and odd basis states simplifies the optimization surface and ensures only basis states of suitable parity contribute to the qDRIVE-optimized state.

**Figure 3:**
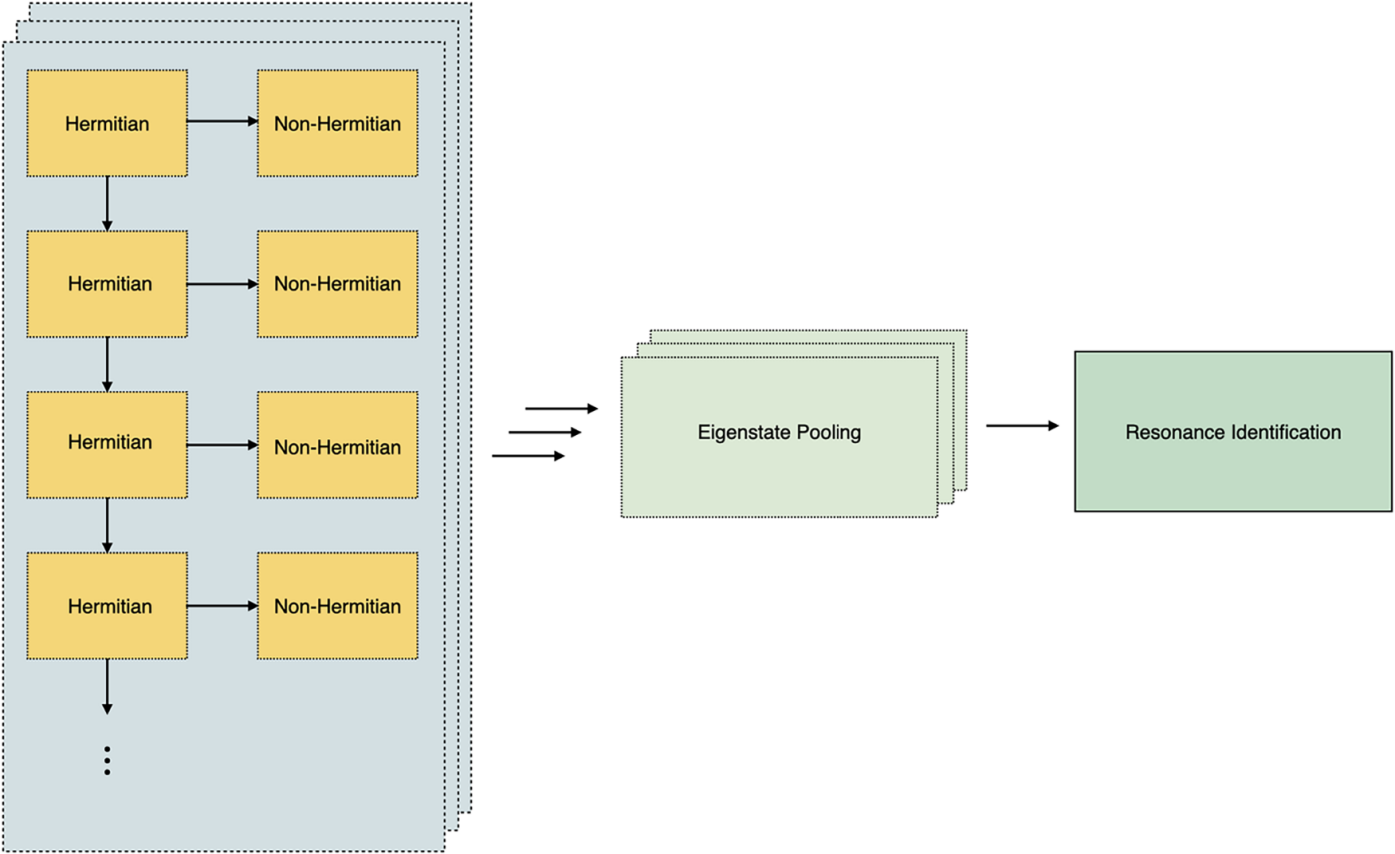
Schematic for implementation of qDRIVE with HTC resources. Each batch (blue rectangles) begins with identification of the ground state of the Hermitian Hamiltonian on an individual classical processor, which spawns both identification of a corresponding eigenstate of the non-Hermitian Hamiltonian on another classical processor and identification of the next excited state on yet another classical processor (yellow rectangles). For each batch, upon completion all resonances are pooled into a single file (lime rectangles), and finally sorted among all batches to identify the result for each resonance with the lowest pseudovariance (green rectangle).

### Near-Term Error Mitigation

To facilitate the implementation of qDRIVE on near-term quantum computers, we employ readout and gate error mitigation in conjunction with special consideration of the identity Pauli word.

#### Readout Error Mitigation

Elementary readout error mitigation is performed via matrix inversion, in which the true statistics of ancilla measurement are inferred from noisy statistics^85–87^ (see also advanced techniques^84^ such as twirled readout error extinction^108,109^ to counteract noise-induced bias). Where the noisy expectation vector *N⃗* is related to the true expectation vector *T⃗* by

11 $$\left[\begin{array}{l} N_{0}\\ N_{1} \end{array}\right]=\left[\begin{array}{l} T_{0}\\ T_{1} \end{array}\right]\left[\begin{array}{ll} p_{00} & p_{01}\\ p_{10} & p_{11} \end{array}\right]$$

where the zeroth and the first element of each vector correspond to the probability the ancilla is measured in the |0⟩ and |1⟩ state, respectively, and where *p*_*ij*_ is the probability the qubit is in true state *i* but measured in state *j* in the presence of noise (determined through system benchmarking^110^); the true expectation vector components are estimated as

12 $$T_{0}=\frac{N_{0}-p_{10}}{p_{00}-p_{10}},T_{1}=\frac{N_{1}-p_{01}}{p_{11}-p_{01}}$$

according to solution of the system of equations given normalization of the true expectation vector *T*_0_ + *T*_1_ = 1 as expected of a classical probability.

#### Gate Error Mitigation

Gate error mitigation is performed via hybrid exponential-linear three-point zero-noise extrapolation (ZNE).^88,90,91^ According to standard ZNE, each circuit result *x*_λ_ is extrapolated to its zero-noise counterpart λ = 0 based on the original result of the circuit λ = 1 and artificially increased-error iterations of the circuit with (λ – 1)/2 repetitions of each gate and its inverse. We thus fit *x*_1_, *x*_3_, and *x*_5_ to the exponential function

13 $$x_{\lambda }\approx A+Be^{C\lambda }$$

via solution of equations for the constants *A*, *B*, *C* to yield the zero-noise extrapolated circuit result^91^

14 $$x_{0}=x_{1}+\frac{x_{1}-x_{3}}{\beta^{2}+\beta },\beta =\sqrt{\frac{x_{3}-x_{5}}{x_{1}-x_{3}}}$$

Importantly, the resulting zero-noise result *x*_0_ is physically realizable $\left(x_{0}\in R\right)$ if and only if (*x*_3_ – *x*_5_)/(*x*_1_ – *x*_3_) > 0 (i.e., where *x*_λ_ is monotonic for the three points considered). We therefore employ physically motivated linear approximations where monotonicity is violated to within statistical significance as detailed in the appendix.

#### Identity Pauli Word

Additionally, we make the simple recognition that the expectation value of the identity Pauli word *I* ^⊗*q*^ is analytically known to be unity

15 $$\left\langle \psi \left|I^{⊗q}\right|\psi \right\rangle =\left\langle \psi |\psi \right\rangle =1$$

for all normalized states ψ, such that the expectation value need not be estimated on the quantum processor. This small point ensures accurate evaluation of the term free of the vicissitudes of readout or gate error, of particular importance for the investigation of chemical Hamiltonians (and associated pseudovariances) for which its associated weight in the Pauli decomposition eq 7 is relatively large.

### Model System

To demonstrate the power of the qDRIVE algorithm, we present the method as applied to a benchmark potential designed to model resonances associated with diatomic predissociation and molecular scattering collisions^62^

16 $$V_{0}\left(x\right)=\left(\frac{1}{2}x^{2}-J\right)e^{-\lambda x^{2}}+J$$

where λ = 0.1 and *J* = 0.8 (arbitrary units by convention), with purely outgoing boundary conditions imposed via a quadratic CAP

17 $$V_{\mathrm{CAP}}\left(x\right)=\{\begin{array}{ll} 0 & \left|x\right|\leq x_{0}\\ -\frac{1}{2}{\left(\left|x\right|-x_{0}\right)}^{2} & \left|x\right|>x_{0} \end{array}$$

where *x*_0_ = 8 au, as illustrated in Figure 4. To aid direct comparison to literature results, we specifically examine the resonance near Re­(*E*_*r*_2__) = 2.13 investigated by Bian et al. with the direct measurement method.^53^ To demonstrate the breadth of the method, we additionally employ qDRIVE to identify the bound state near *E_b_* = 0.502 and the narrower resonance near Re­(*E*_*r*_1__) = 1.42. Simulations shown here are performed for a Hamiltonian implemented in position-space representation in the domain *x* ∈ [−*x*_max_,*x*_max_] with *x*_max_ = 10 and 2^12^ gridpoints, a kinetic energy operator computed according to the second-order finite differencing gradient, and a basis set comprising of 2^*q*^ sinusoidal basis states of length *L* = 2*x*_max_

18 $$\phi_{k}\left(x\right)=\sqrt{\frac{2}{L}}\sin\left(\frac{w\pi }{L}\left(x-\frac{L}{2}\right)\right)$$

of either even *w* = 1, 2, 3, ... or odd *w* = 2, 4, 6, ... symmetry.

**Figure 4:**
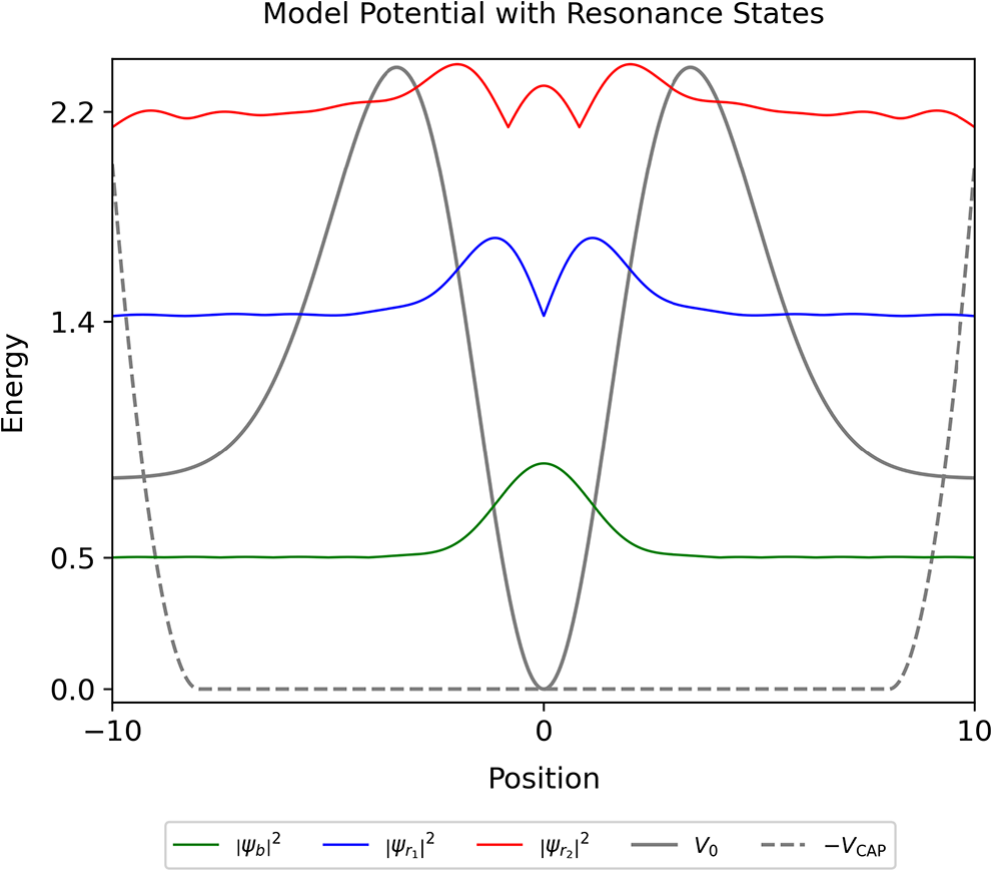
Benchmark potential energy surface *V*_0_ (solid gray line), which supports a bound state ψ_*b*_ and two resonances ψ_*r*_1__ and ψ_*r*_2__ (solid green, blue, and red lines, probability density shown), here termed the bound state, first resonance, and second resonance, respectively, shown as computed via the established classical CAP method of exact diagonalization where purely outgoing boundary conditions are imposed by a complex absorbing potential *V*_CAP_ (dashed gray line, shown as −*V*_CAP_). Each state’s probability density is vertically shifted by the value of the real part of its energy Re­(*E*).

### Quantum Simulation and Experimental Tests

All codes for quantum simulation and experimental tests have been made available publicly under an open-source license from Github^111^ using IBM Qiskit.^112^ The statevector simulator is used to examine qDRIVE in the absence of noise; the Aer simulator, in the presence of only statistical shot noise; and a custom simulator, in the presence of statistical shot noise, readout noise, and gate noise. IBM Falcon r4T processors are used to examine the efficacy of real quantum computers for the most quantum-processor-intensive stage of qDRIVE (namely, pseudovariance minimization).

In simulations, where statistical shot noise is incorporated, *n* = 10^5^ shots are taken per quantum circuit. An additional order of magnitude of shots is employed for final comparison of results between runs. Where readout and gate error are included, the noise model for thermal relaxation at time *t* incorporates both the generalized amplitude damping channel Kraus matrices

19 $$K_{0}=\sqrt{1-p}\left[\begin{array}{ll} 1 & 0\\ 0 & \sqrt{1-\gamma_{1}} \end{array}\right],K_{1}=\sqrt{1-p}\left[\begin{array}{ll} 0 & \sqrt{\gamma_{1}}\\ 0 & 0 \end{array}\right]$$

20 $$K_{2}=\sqrt{p}\left[\begin{array}{ll} 1 & 0\\ 0 & \sqrt{1-\gamma_{1}} \end{array}\right],K_{3}=\sqrt{p}\left[\begin{array}{ll} 0 & \sqrt{\gamma_{1}}\\ 0 & 0 \end{array}\right]$$

21 $$\gamma_{1}=1-e^{-t/T_{1}}$$

where *p* is the equilibrium excited state population and *T*_1_ is the generalized amplitude damping relaxation time, and the phase damping model Kraus matrices

22 $$K_{0}=\left[\begin{array}{ll} 1 & 0\\ 0 & \sqrt{1-\gamma_{2}} \end{array}\right],K_{1}=\left[\begin{array}{ll} 0 & 0\\ 0 & \sqrt{\gamma_{2}} \end{array}\right]$$

23 $$\gamma_{2}=1-e^{-t/T_{2}}$$

where *T*_2_ is the phase damping relaxation time. The local one- and two-qubit depolarizing error is then described by the error channel

24 $$E\left(\rho \right)=\left(1-p_{d}\right)\rho +p_{d}\mathrm{Tr}\left[\rho \right]\frac{I}{2^{d}}$$

for *d*-qubit gate error probability *p*_*d*_. In the custom simulator model of IBM Torino, qubit and processor specifications are hard-coded to approximate the contemporary properties of the quantum processor, including system basis gates and qubit-specific one- and two-qubit gate errors, readout errors, *T*_1_/*T*_2_ times, and gate times. To reduce simulation cost, only five qubits of the full IBM Torino system are simulated to accommodate a maximal four-qubit Ansatz and a single ancilla. Quantum processors with specifications beyond those of IBM Torino are simulated using the same custom simulator with a gate noise reduction factor applied to the one- and two-qubit gate error probabilities *p*_1_ and *p*_2_ and a qubit longevity factor that represents the order of magnitude of the *T*_1_ relaxation time where the leading digit of *T*_1_ and the ratio between the *T*_1_ and *T*_2_ relaxation times is held fixed. For example, where the IBM Torino relaxation times *T*_1_ and *T*_2_ are approximated as 70 μs and 50 μs, respectively, a qubit longevity factor of 100 μs implies relaxation times *T*_1_ and *T*_2_ of 700 and 500 μs, respectively. Infinite qubit longevity factors correspond to the absence of thermal relaxation.

In experimental tests on real quantum processors, the most computationally intensive stage of qDRIVE for the quantum processor, namely, pseudovariance minimization, is tested on five-qubit IBM Falcon r4T quantum processors Quito, Belem, and Lima. Device specifications, including qubit relaxation times and gate error rates, are provided for reference in Table 1. To isolate error due to pseudovariance minimization from that of variational quantum deflation (VQD), Ansatz parameters are randomly initialized in the domain θ_*i*_*o*__ ∈ [−π, π] where *o* ∈ [1, *m*]. A two-local circuit with *R*_*x*_ and *R*_*y*_ rotation gates is employed with full entanglement between qubits provided by CNOT gate layers in order to balance Ansatz expressibility with quantum processor cost where applicable. Energies are computed for one- and two-qubit Hamiltonians represented in a sinusoidal basis using a shot number of *n* = 10^4^ on the quantum processor and COBYLA^104^ (tolerance 10^–3^, maximum iteration number 15) on the classical processor. Given limited quantum processing resources, real quantum computer tests are automatically executed on the least occupied quantum processor and in such tests no error mitigation methods are employed.

**Table 1:** Specifications of IBM Falcon r4T Quantum Processor Devices (Version in Parentheses) Employed in Experiments, as of September 14, 2023, from IBM Qiskit^112^ ^a^

				error					
device	qubit	*T*_1_	*T*_2_	readout	*I*	$\sqrt{X}$	*X*	CNOT	gate time
Quito (1.1.45)	0	112.5	139.23	3.340	2.437	2.437	2.437	0_1:0.03884	0_1:234.667
	1	106.41	101.49	3.640	2.726	2.726	2.726	1_3:0.00958	1_3:334.222
								1_2:0.01122	1_2:298.667
								1_0:0.03884	1_0:270.222
	2	63.66	37.37	7.640	3.474	3.474	3.474	2_1:0.01122	2_1:263.111
	3	58.45	18.14	3.920	2.713	2.713	2.713	3_4:0.01469	3_4:277.333
								3_1:0.00958	3_1:369.778
	4	70.44	131.29	4.05	2.767	2.767	2.767	4_3:0.01469	4_3:312.889
Belem (1.2.12)	0	116.9	135.85	2.740	2.104	2.104	2.104	0_1:0.01478	0_1:810.667
	1	93.12	92.11	1.960	4.920	4.920	4.920	1_3:0.00669	1_3:440.889
								1_2:0.00586	1_2:419.556
								1_0:0.01478	1_0:775.111
	2	58.22	47.91	1.65	1.976	1.976	1.976	2_1:0.00586	2_1:384.00
	3	88.4	94	2.63	2.721	2.721	2.721	3_4:0.00908	3_4:526.222
								3_1:0.00669	3_1:405.333
	4	58.15	45.07	1.73	5.656	5.656	5.656	4_3:0.00908	4_3:490.667
Lima (1.0.52)	0	113.67	160.08	1.96	7.567	7.567	7.567	0_1:0.00662	0_1:305.778
	1	93.43	131.71	1.740	5.44	5.44	5.44	1_0:0.00662	1_0:341.333
								1_3:0.01152	1_3:497.778
								1_2:0.00695	1_2:334.222
	2	69.01	99.69	2.06	7.334	7.334	7.334	2_1:0.00695	2_1:298.667
	3	59.08	91.58	3.85	2.367	2.367	2.367	3_4:0.01476	3_4:519.111
								3_1:0.01152	3_1:462.222
	4	16.13	16.17	4.43	6.514	6.514	6.514	4_3:0.01476	4_3:483.556

a Values are reported in microseconds for relaxation times *T*_1_ and *T*_2_, nanoseconds for two-qubit gate times, and with implied multiplicative factors of 10^–2^, 10^–4^, 10^–4^, 10^–4^, and 10^–3^ for readout assignment, *I*, $\sqrt{X}$ , *X*, and CNOT error rates, respectively.

Results are reported in terms of energies computed at the level of quantum simulation or quantum computation used and eigenstate probability densities are visualized where relevant with the statevector simulator. To evaluate the success of qDRIVE, qDRIVE’s accuracy, robustness, and computational cost are compared to that of two established classical resonance identification techniques: (1) the established classical CAP resonance identification method in which resonances are determined via exact diagonalization of *H*_N_ = *H*_H_ + i*V*_CAP_,^35–39^ and (2) the established classical complex scaling method in which resonances are determined via θ trajectories of the complex-scaled Hamiltonian.^40,41,53^ Classical CAP method results are presented for identical basis sets to that of qDRIVE for direct comparison, and classical complex scaling method results are presented for a basis set of five orthogonalized Gaussian basis states χ_*k*_(α) = exp­(−α_*k*_ *x* ^2^) with α_*k*_ = 0.65·0.45^*k*^ where *k* = 0, 1, 2, 3, 4 for comparison to the literature solution of ref 53.

### Results

The interlaced quantum computing/HTC qDRIVE approach was found to accurately identify all resonance energies considered for the benchmark resonance model of molecular predissociation eq 16 as compared to established classical resonance identification techniques. As shown in Table 2, the error of the bound-state energy *E*_*b*_, the first-resonance energy *E*_*r*_1__, and the second-resonance energy *E*_*r*_2__ relative to the established classical CAP method results was found to remain below 1% in all statevector simulations for two- to four-qubit Ansätze. Additionally, the relative error was observed to be as low as *O*(10^–5^%) for the highest qubit-number statevector simulation considered, indicative of the success of the method in the absence of shot noise and readout and gate error. In experiments with the Aer simulator that included shot noise, the relative error remained below 1% in all simulations with the exception of a relative error of 2.8% for *E*_*r*_2__ where a three-qubit Ansatz was employed, which was also associated with larger componentwise error of Im­(*E*_*b*_) and Im­(*E*_*r*_1__). As expected, in custom simulations of IBM Torino that included readout and gate error, the relative error was larger but still in the vicinity of classical CAP method results, with an error as low as 0.91% for *E*_*r*_1__ with a two-qubit Ansatz and as high as 35% for the same energy *E*_*r*_1__ with a three-qubit Ansatz, which suggests qDRIVE may be already implemented as specified with existing quantum computing systems where the error tolerance is acceptable. qDRIVE results for the second resonance energy *E*_*r*_2__ were also found to be accurate relative to the literature classical complex scaling method result from ref 53 of *E* = 2.1265 – 0.0203i. As expected, the accuracy of qDRIVE relative to the established classical complex scaling resonance identification technique was found to increase as the basis set size increased, with a relative error of 10.0%, 10.5%, and 3.5% for two-qubit statevector, Aer, and Torino, respectively; 0.8%, 1.7%, and 2.6% for three-qubit statevector, Aer, and Torino, respectively; and 0.9% for four-qubit statevector. Note the qDRIVE simulations were also found to be highly robust relative to established classical resonance identification techniques, as the bound and resonance states were identified in 100% of the quantum simulations for the number of batch runs considered, in full agreement with the robustness of classical CAP and classical complex scaling methods.

**Table 2:** (a) Bound and Resonance State Energies *E*_*b*_, *E*_*r*_1__, and *E*_*r*_2__ Computed According to qDRIVE for a *q*-Qubit Ansatz via the Quantum Simulators (Sim.) Statevector, Aer, and IBM Torino, as Compared to the Result of the Classical CAP Method According to (b) Percent Relative Error $E=\left|z^{\prime }-z\right|/\left|z\right|$

*q*	sim.	*E*_*b*_	*E*_*r*_1__	*E*_*r*_2__
2	Classical CAP Method	0.623 – 2.63·10^–3^ i	1.61 – 4.15·10^–2^i	2.36 – 5.83·10^–3^i
	Statevector	0.623 – 2.78·10^–3^i	1.61 – 4.26·10^–2^i	2.34 – 1.22·10^–2^i
	Aer	0.619 – 2.97·10^–3^i	1.61 – 3.97·10^–2^i	2.35 – 7.13·10^–3^i
	Torino	0.685 – 2.37·10^–3^i	1.61 – 5.62·10^–2^i	2.20 – 1.29·10^–2^i
3	Classical CAP Method	0.505 – 2.02·10^–5^i	1.43 – 1.61·10^–4^i	2.15 – 2.04·10^–2^i
	Statevector	0.504 – 2.48·10^–5^i	1.43 – 1.44·10^–4^i	2.14 – 1.00·10^–2^i
	Aer	0.505 + 2.43·10^–4^i	1.44 + 2.60·10^–4^i	2.09 – 2.64·10^–2^i
	Torino	0.562 + 2.22·10^–3^i	1.92 – 8.85·10^–2^i	2.18 – 1.09·10^–2^i
4	Classical CAP Method	0.502 – 9.98·10^–11^i	1.42 – 3.60·10^–5^i	2.12 – 1.18·10^–2^i
	Statevector	0.502 – 1.23·10^–7^i	1.42 – 4.03·10^–5^i	2.13 – 4.63·10^–4^i

*q*	sim.	$E_{b}$	$E_{r_{1}}$	$E_{r_{2}}$
2	Statevector	0.02%	0.07%	0.89%
	Aer	0.64%	0.11%	0.43%
	Torino	9.95%	0.91%	6.79%
3	Statevector	0.20%	0.0012%	0.67%
	Aer	0.05%	0.70%	2.80%
	Torino	11.30%	34.82%	1.46%
4	Statevector	0.000024%	0.00030%	0.71%

Results of the real quantum computer experiments, shown in Table 3, indicate that pseudovariance minimization successfully yielded complex energies near eigenvalues of the Hamiltonian. Both eigenvalues of the one-qubit Hamiltonian were identified, with a relative error of 17.0% and 5.1% relative to the classical CAP method result. The two complex energies of the two-qubit Hamiltonian with a real part closest to that of the scattering *r*_1_ and *r*_2_ resonances observed for larger basis sets were likewise measured with 30.5% and 8.9% relative error relative to the classical CAP method of exact diagonalization of the same Hamiltonian, respectively. As expected, the resonance energies were therefore within the vicinity of but with a higher degree of relative error than statevector, error-mitigated Aer, and error-mitigated simulated IBM Torino resonance energies for the system examined in Table 2.

**Table 3:** Energies of Selected States Computed via qDRIVE Pseudovariance Minimization of a Randomly Initiated *q*-Qubit Ansatz on IBM Falcon r4T Quito, Lima, and Belem Processors, with Percent Relative Error E Calculated Relative to the Classical CAP Method of Exact Diagonalization of the Corresponding Hamiltonian^a^

*q*	classical CAP method	IBM Falcon r4T	E
1	0.55 + 0.0000i	0.54 + 0.093i	17.0%
	2.22 + 0.0000i	2.11 + 0.0248i	5.1%
2	1.56 + 0.0698i	1.39 – 0.3745i	30.5%
	2.26 – 0.0034i	2.06 – 0.007i	8.9%

a Note the two-qubit classical CAP method values are distinct from those of Table 2 as a combined even/odd basis set is employed.

qDRIVE was also found to successfully produce probability densities consistent with classical CAP method results for all resonances considered, as shown in Figure 5 for a three-qubit Ansatz. Probability densities from statevector simulations closely agreed with classical CAP method results for the bound state and first resonance, with a higher discrepancy in the second resonance that may be attributable to the underlying deflation procedure given the high-lying nature of its corresponding Hermitian Hamiltonian eigenstate. Probability densities from Aer simulations were found to be accurate in the internal region of the potential, with a lower degree of accuracy than statevector, as expected due to the additional consideration of statistical noise. Consistent with the impact of further readout and gate noise, the greatest discrepancy between the qDRIVE-optimized and classical-CAP-method probability densities was observed for the custom simulations of IBM Torino; nonetheless, overall trends of localization of the bound state and resonances in the potential’s internal region and reduction in the outer region were preserved.

**Figure 5:**
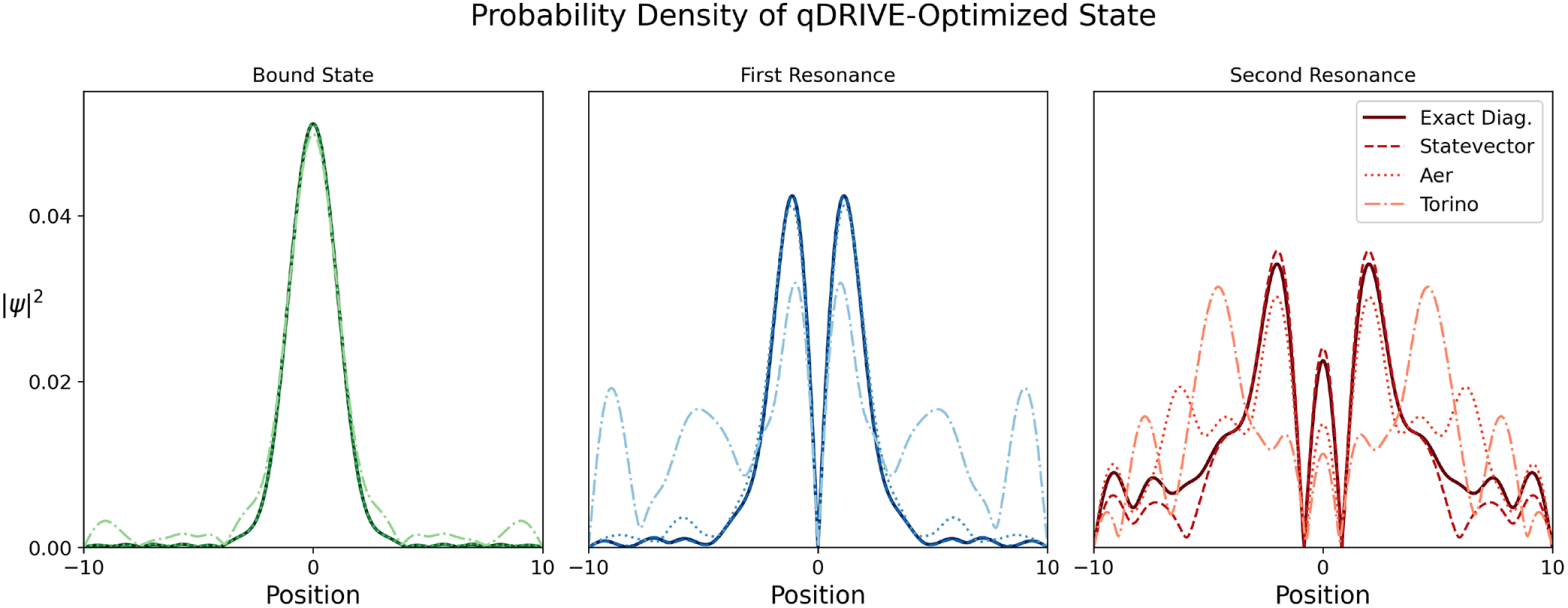
Probability density |ψ|^2^ visualized in position space corresponding to the qDRIVE-optimized three-qubit Ansatz for the statevector (dashed lines), Aer (dotted lines), and Torino (dashed-dotted lines) simulators as compared to classical CAP method/exact diagonalization (solid lines) for the (left) bound state (green lines), (center) first resonance (blue lines), and (right) second resonance (red lines).

Increased gate noise reduction and qubit longevity factors were associated with lower pseudovariance and fidelity error of the qDRIVE-optimized resonances, as shown in Figures 6 and 7. Both the pseudovariance and the fidelity error were found to approach their optimal value of zero as the gate noise reduction factor increased from 10^0^ to 10^4^ and the qubit longevity increased from 10^1^ to ∞ μs, with fluctuations in keeping with the statistical nature of the simulations. An increase in the maximal value of the pseudovariance was also observed for states corresponding to higher-lying eigenstates of the Hermitian Hamiltonian in keeping with errors introduced by deflation.

**Figure 6:**
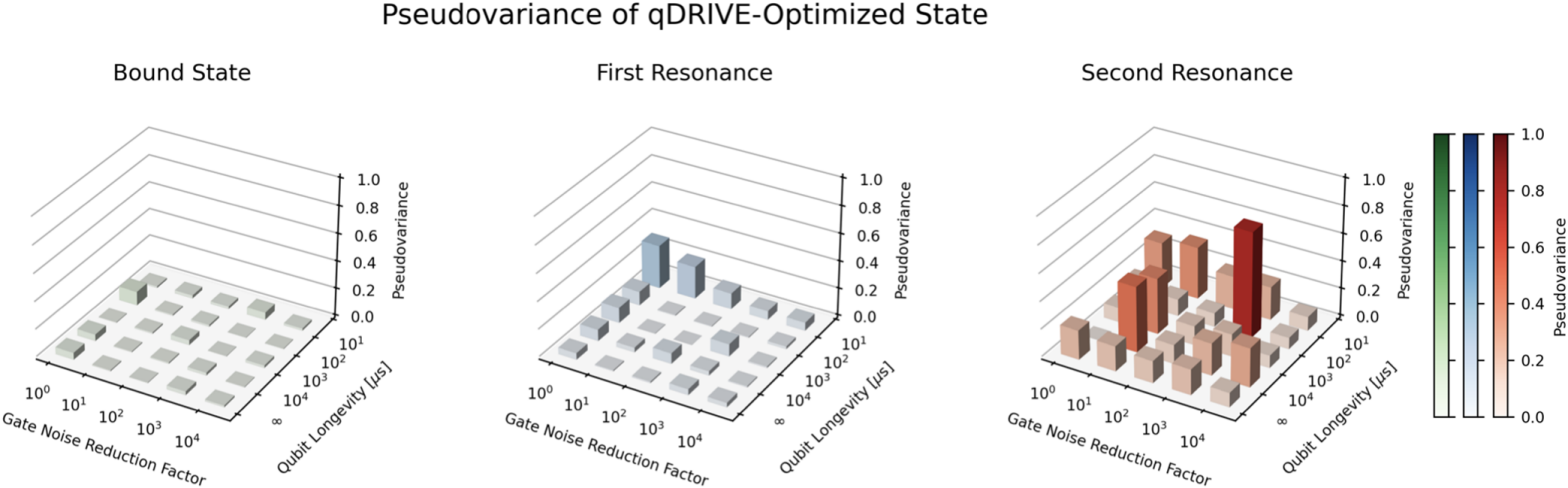
Pseudovariance eq 2 of the (left) bound state, (center) first resonance, and (right) second resonance as determined by qDRIVE with a three-qubit Ansatz as a function of the gate noise reduction and qubit longevity factors.

**Figure 7:**
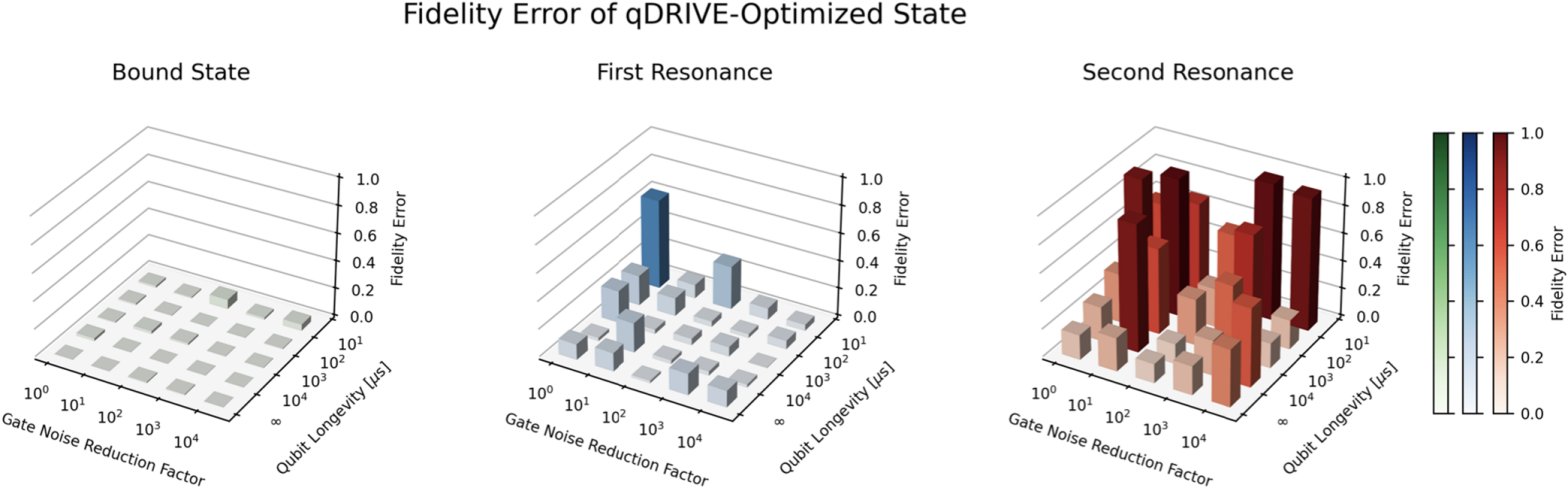
Fidelity error of the simulated qDRIVE-optimized (left) bound state, (center) first resonance, and (right) second resonance ψ_Sim_ as compared to the classical CAP method/exact diagonalization result ψ_Exact_, 1–|⟨ψ_Sim_|ψ_Exact_⟩|^2^, for increasing gate noise reduction and qubit longevity factors.

Notably, convergence of the complex energies to their classical CAP method values was evident even where the pseudovariance and fidelity errors were far from their optimal zero values, as depicted in Figures 8, 9, and 10 for a three-qubit Ansatz. As shown in Figure 8, the real part of the qDRIVE-optimized bound-state, first-resonance, and second-resonance energies closely agreed with the classical CAP method value for all gate noise reduction and qubit longevity factors considered, with imaginary parts with an absolute error within 0.1 regardless of gate noise for qubit longevity factors of at least 1000 μs, on the order of the millisecond times recently achieved on advanced 2D transmon qubits.^113^ The bound-state energy specifically was found to be nearly indistinguishable from the classical CAP method result at the resolution shown without magnification for qubit longevity factors of at least 10 μs, namely, on the order of IBM Torino relaxation times. As emphasized in Figure 9, the bound-state and first-resonance energies’ imaginary parts were accurate to within an absolute error of 0.1 for gate noise reduction factors of at least 10^0^ and qubit longevity factors of at least 10 μs, both of which are accessible with current IBM Torino systems. Consistent with trends in the pseudovariance and fidelity error, higher errors in the real and imaginary parts of the energy were observed for the second resonance associated with a higher-lying eigenstate of the Hermitian Hamiltonian. As shown in Figure 10, weaker nonmonotonic convergence of the imaginary part of the qDRIVE-optimized energy to classical CAP method/exact diagonalization results was observed for the second resonance as the gate noise reduction factor increased, a correlation between repeated deflation and error emergence that suggests possible benefits of the future injection into qDRIVE of higher-accuracy VQD variants (such as the tangent-vector VQE method^114^) or variational algorithms for high-lying eigenstate identification (such as adaptive VQE-X^95^).

**Figure 8:**
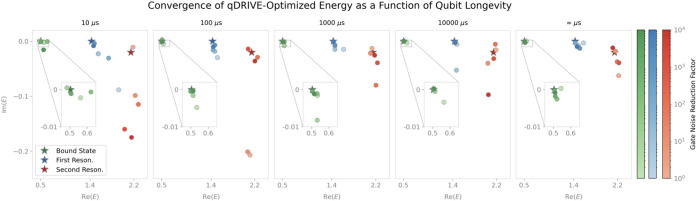
Approach of the bound-state (with magnification), first-resonance, and second-resonance energy (circles; green, blue, and red, respectively) to the corresponding classical CAP method/exact diagonalization energy (stars) with increased qubit longevity. Darker colors indicate higher gate noise reduction factor.

**Figure 9:**
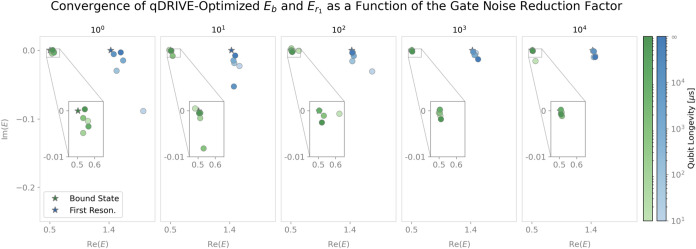
Progression of the energy of the bound state and first resonance (green and blue circles, respectively) toward the corresponding classical CAP method/exact diagonalization energy (green and blue stars, respectively) as the gate noise reduction factor increases. Darker colors indicate longer qubit longevity.

**Figure 10:**
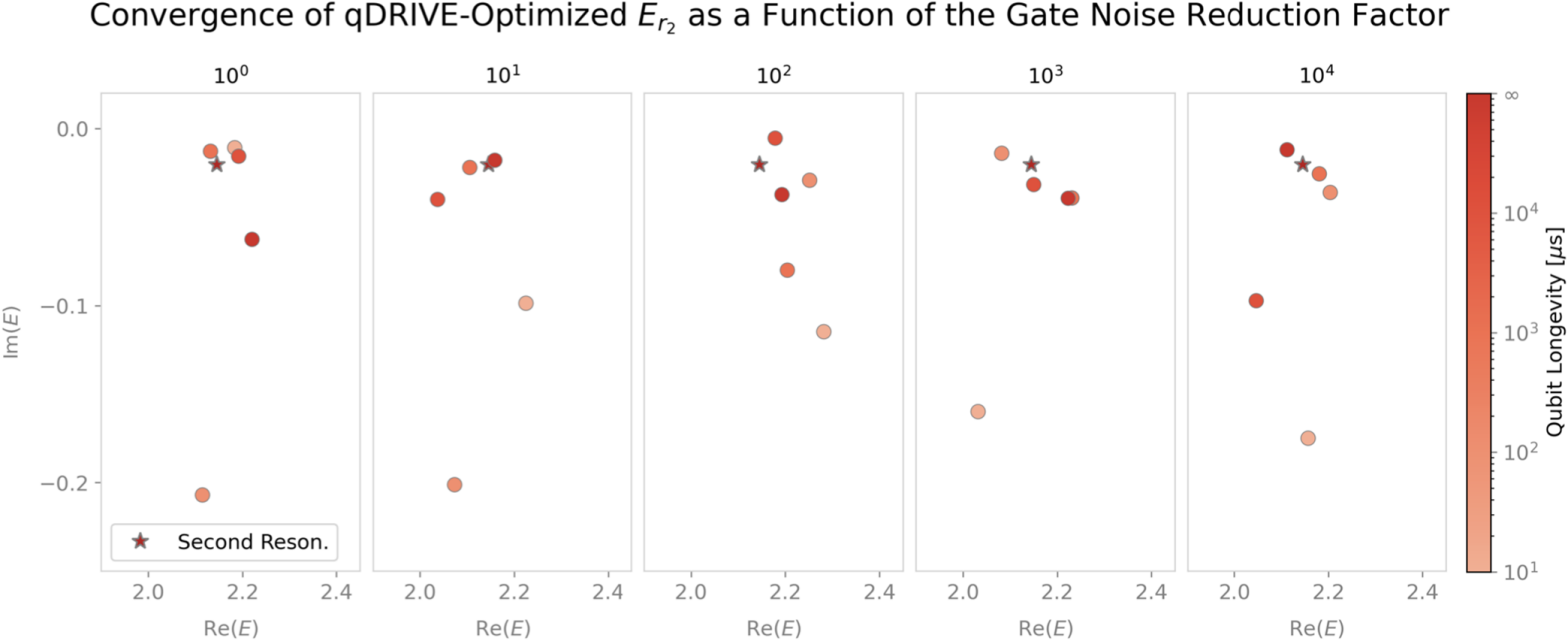
As in Figure 9, for the second resonance.

## Discussion and Conclusion

The success of the proposed qDRIVE algorithm points to the potential efficiency and efficacy of a hybrid quantum computing/high-throughput computing (HTC) approach to chemistry. qDRIVE’s ability to identify molecular resonance energies and wave functions in a benchmark potential on simulated noisy intermediate scale quantum (NISQ) computers invites its implementation on today’s quantum processors. Its projected accuracy for more advanced quantum processors suggests the algorithm may offer yet improved accuracy as quantum processors continue to develop. And, more broadly, qDRIVE’s success serves as a prototype for a wider family of heterogeneous quantum computing algorithms.

Making qDRIVE fully practical for systems beyond the small-scale systems considered here will require addressing the issue of Hamiltonian mapping. It is well-known that grid-based mappings, such as the method used here to represent the benchmark potential, feature a storage cost that grows exponentially with the grid size.^56,115^ Molecular electronic resonances^32,116^ may be able to circumvent this problem given the existence of alternative, scalable Hamiltonian mappings for electronic structure problems^117^ such as the renowned Jordan–Wigner^118^ and Bravyi–Kitaev^119^ fermion-qubit mappings. Additionally, where decompositions of the Hamiltonian and its products result in a large number of expectation values to be measured, a variety of methods currently under development may reduce the number of expectation values that need to be computed, and therefore reduce the overall cost of qDRIVE’s implementation. Classical shadow tomography,^120–122^ for example, allows for the reconstruction of unmeasured expectation values based on the “shadow” of a smaller subset of measured values, and the method of commuting strings^123^ allows for large numbers of expectation values to be computed with comparatively fewer quantum circuits.

An adaptive Ansatz approach offers a pathway to the extend qDRIVE to larger-scale systems. qDRIVE’s observed ability to accurately identify resonance energies and wave functions suggests the three-layer efficient SU(2) Ansatz is sufficient for analysis of the benchmark molecular system of interest; however, a key question is how to maintain this expressibility and efficiency upon the addition of parameters and entanglement between large numbers of qubits. One possibility is ADAPT-VQE (Adaptive Derivative-Assembled Pseudo-Trotter Ansatz Variational Quantum Eigensolver), which has been seen to be a highly effective means to improve the scalability of VQE.^124^ By optimizing both the parameters and form of the Ansatz, a variant of ADAPT-VQE has successfully enabled VQE on 127-qubit processors, including a 100-qubit study of the lattice Schwinger model.^125^

The contracted quantum eigensolver (CQE)^15,94,126,127^ provides another pathway to expand qDRIVE to larger system sizes. As in ADAPT-VQE, CQE possesses the ability to modify an Ansatz until it suits the quantum system under study. However, unlike ADAPT-VQE, CQE’s two-body Ansatz construction method never calls for parameter reoptimization. This distinction, along with the structure of the contracted Schrödinger equation on which CQE relies, allows CQE to readily produce analytic gradients of the energy with respect to the Ansatz parameters. Thus, whereas ADAPT-VQE typically relies on gradient-free classical optimization algorithms such as COBYLA, CQE can capitalize on efficient gradient-based classical optimization algorithms that offer important cost savings where quantum resources remain scarce.

In established classical resonance identification techniques, the computational cost generally scales directly with the size of the Hamiltonian matrix. For example, the matrix diagonalizations frequently used in classical CAP and complex scaling methods^62,128^ scale as *O*(*N* ^3^). In contrast, the computational cost of qDRIVE can scale independently of the Hamiltonian matrix size. qDRIVE’s variational hybrid quantum-classical computing approach is expected to give qDRIVE a computational complexity akin to that of VQE,^3^ namely, the cost of identifying of a single state in qDRIVE is expected to scale as *O*(|*C*_max_|^2^ *tp* ^–2^), where *C*_max_ is the largest-magnitude coefficient of the Pauli decomposition eq 7, *t* is the number of terms in said decomposition, and *p* is the desired precision.[Fn fn2] The cost comparison therefore suggests the value of a search for possible advantage in the large system limit, with the caveat that the quantum utility of variational quantum algorithms remains a topic of continued heated debate.^57,58^

qDRIVE’s success invites the further development of integrated quantum computing and HTC approaches. The interconnection of high-performance computing (HPC) resources and quantum computers is an emerging hot topic in quantum information science, and much focus has been placed on distributed quantum computing systems based either on multiple quantum processing units working in concert or heterogeneous computing systems consisting of central processing units (CPUs), graphical processing units (GPUs), and quantum processing units (QPUs).^68–70^ The accomplishments here of qDRIVE for molecular resonance identification, paired with the accomplishments of HTC in classical scientific computing for applications ranging from biochemical network^129^ to high-energy particle physics^130^ simulations, open the door to a powerful strategy of executing computational tasks in parallel yet taking advantage of asynchronicity.

## Footnotes

1 Note an outlier, the Quantum Annealer Eigensolver,^24^ which is specifically designed for use on quantum annealing (e.g., D-Wave) systems.^51^

2 Note identification of additional states with qDRIVE is expected to indirectly depend on the matrix size due to the need for successive deflations. However, this dependence may be removed via substitution of the VQD routine with a suitable alternative for high-lying eigenstate identification. Likewise, although at its face the number of terms appears to scale with the matrix size as *t* ∝ *N* ^2^, the number of terms is generically reducible to the number of groups of commuting Pauli strings according to the method of commuting strings^123^ and in many cases the number of terms is naturally significantly lower due to fortuitous construction of the Hamiltonian.

## Appendix: Edge Cases of Exponential Zero-Noise Extrapolation

In edge cases for which exponential three-point ZNE produces a physically unrealizable zero-noise result $x_{0}\notin R$ , linear zero-noise extrapolation is employed to mitigate gate error as follows:

Where *x*_3_ < *x*_1_ < *x*_5_, contrary to the physical expectation that noise increases monotonically with λ, the optimal least-squares curve with

25
$$A=\left(x_{1}+x_{3}\right)/2,B\to 0_{\pm },C\to \infty$$

remains nearly constant between *x*_1_ and *x*_3_ and increases steeply to meet *x*_5_ exactly; we thus exclude *x*_5_ as an outlier and employ the zero-noise estimate

26
$$x_{0}=\left(x_{1}+x_{3}\right)/2$$

Where *x*_1_ < *x*_5_ < *x*_3_, since the optimal least-squares curve with

27
$$A=\left(x_{1}+x_{3}\right)/2,B\to \pm \infty ,C\to -\infty$$

yields the physically unreasonable estimate *x*_0_ = ±∞, and since *x*_5_ is expected to give rise to more error than *x*_3_ where the system is not fully depolarized; we extrapolate *x*_1_ and *x*_3_ to *x*_0_ via the linear fit *x*_λ_ ≈ *A*λ + *B* such that the zero-noise estimate is

28
$$x_{0}=\frac{3x_{1}-x_{3}}{2}$$

Where *x*_3_ ≈ *x*_5_, in order to avoid the instability *x*_0_ → ±∞ as *x*_3_ → *x*_5_, we evaluate the statistical significance between *x*_3_ and *x*_5_ according to a two-sample *z*-test at the α = 0.05 level (for which statistical evidence corresponds to a *z*-score of |*z*| > 1.96). Given that measurement of the ancilla projects the qubit onto either the |0⟩ or |1⟩ state, the *n*-shot standard deviation takes the form of a binomial distribution

29
$$s=\sqrt{\frac{p\left(1-p\right)}{n}}$$

which entails a joint standard deviation of

$$s_{\mathrm{joint}}=\sqrt{\frac{x_{3}\left(1-x_{3}\right)+x_{5}\left(1-x_{5}\right)}{n}}$$

and associated z-score of

$$z=\frac{x_{3}-x_{5}}{\sqrt{\frac{x_{3}\left(1-x_{3}\right)+x_{5}\left(1-x_{5}\right)}{n}}}$$

We therefore we use the exponential fit estimate eq 14 if

$$\frac{\left|x_{3}-x_{5}\right|}{\sqrt{\frac{x_{3}\left(1-x_{3}\right)+x_{5}\left(1-x_{5}\right)}{n}}}>1.96$$

and the linear fit eq 28 otherwise.

Where *x*_1_ ≈ *x*_5_, the estimate *x*_0_ becomes undefined (given the existence of two equally applicable but mutually inconsistent least-squares curves eqs 25 and 27), such that we employ the average estimate for *x*_3_ < *x*_1_ < *x*_5_ eq 25 and *x*_1_ < *x*_5_ < *x*_3_ eq 27

30
$$x_{0}=x_{1}$$

Where *x*_1_ ≈ *x*_3_ ≈ *x*_5_, the least-squares curve is

31
$$x_{1}=x_{3}=x_{5}=A+B,C=0$$

such that we estimate *x*_0_ as the constant value

32
$$x_{0}=x_{1}=x_{3}=x_{5}$$
